# Sexual and reproductive health perceptions and practices as revealed in the sexual history narratives of South African men living in a time of HIV/AIDS

**DOI:** 10.1080/17290376.2014.985701

**Published:** 2014-12-12

**Authors:** Erin Stern, Asta Rau, Diane Cooper

**Affiliations:** ^a^ Ph.D., is at the Women's Health Research Unit, School of Public Health, University of Cape Town, Cape Town, South Africa; ^b^ Ph.D., is at Center for Health Systems Research & Development, University of the Free State, Bloemfontein, South Africa; ^c^ Ph.D., is at the Women's Health Research Unit, School of Public Health, University of Cape Town, Cape Town, South Africa

**Keywords:** masculinities, HIV prevention, narratives, sexual and reproductive health, South Africa, les masculinités, prévention du HIV, les récits, la santé sexuelle et génésique, Afrique du Sud

## Abstract

The frequent positioning of men's sexual risk-taking as driving the HIV/AIDS epidemic in South Africa has triggered interest in men's sexual and reproductive health (SRH) perceptions, attitudes, and practices. Much research, however, presents men as a homogenous group, and focuses on the quantifiable aspects of male sexual behaviors, providing an inadequate basis for understanding men's SRH needs and addressing the gendered aspects of HIV prevention. This study used sexual history narratives to yield more nuanced and contextualized understandings of male sexuality as it relates to SRH. Fifty sexual life history individual interviews and 10 focus-group discussions (FGDs) with men, as well as 25 sexual life history interviews with women, were conducted with participants purposively sampled from three age categories: (18–24, 25–55, and 55+ years), a wide range of cultural and racial backgrounds, and in urban and rural sites across 5 provinces in South Africa. Interviews and FGDs elicited stories of participant's early knowledge of sex and sexual experimentation and then explored sexual relationships and experiences in adulthood—including engagement with HIV risks and SRH management. The data were analyzed using a thematic approach. Many male participants conformed to dominant norms of masculinity associated with a high risk of sexually transmitted infections including HIV, such as having regular unprotected sex, reluctance to test for HIV, and poor SRH-seeking behaviors. Yet, the narrative accounts reveal instances of men taking steps to protect their own SRH and that of their partners, and the complex ways in which hegemonic gender norms influence men and women's SRH. Ultimately, the study points to the value of sexual biographies for gaining a deeper understanding of male sexuality, and the social structures, meanings, and experiences that underlie it. Such insights are critical to more effectively engaging men in HIV prevention efforts.

## Résumé

l'idée reçue sur le comportement sexuel risqué des hommes comme principal instigateur du virus du SIDA en Afrique du Sud suscite un vif intérêt sur les perceptions de reproduction, de pratiques et attitudes sexuelles auprès de cette catégorie de la population. Cependant, une grande partie de la recherche sur ce thème, présente les hommes comme un groupe homogène en cherchant à quantifier certains aspects de leurs comportements sexuels. Ce qui génère une base de données inadéquate pour comprendre leurs besoins sur les aspects de genre dans la prévention du virus du SIDA. Cette étude se base sur le contexte sexuel historique de la narration pour une compréhension plus nuancée et contextualisée de la sexualité de l'homme en fonction de la santé reproductive de l'homme. Cinquante entrevues avec des hommes sur leur histoire sexuelle, dix groupes focaux avec des hommes, et vingt cinq entrevues additionnels avec des femmes sur leur histoire sexuelle ont été menées avec des participants recrutés en fonction d'un échantillonnage de trois catégories d'âge (18–24, 25–55, and 55+), et de groupes culturels et raciaux divers. Vivant en milieux urbains et ruraux dans cinq provinces de l'Afrique du Sud. Pourtant, le récit historique démontre la diversité de la sexualité de l'homme , intégrant des hommes qui prennent des mesures de protection dans leur relation sexuelle reproductive, et celles de leurs partenaires. En conclusion, l'étude démontre la valeur narrative de l'histoire sexuelle pour générer une compréhension plus profonde de la sexualité masculine , les structures sociales , les significations et expériences qui l'étayent . Tels sont les aperçus qui permettront de mieux engager les hommes dans la prévention du HIV.

## Introduction

This paper focuses on how norms and constructions of masculinity influence men's sexual and reproductive health (SRH) practices and health-seeking behaviors. In this regard, most research to date has tended to ‘conceive of men as one category and to impute one idea of what it means “to be a man” to South Africans’ (Morrell, Jewkes & Lindegger 2012:14). Men whose perceptions and practices challenge dominant gender norms are often neglected in such analyses (Peacock, Khumalo & McNab 2006). Our aim is to yield more nuanced and contextualized understandings of male sexuality as it relates to SRH in a variety of geographical and social contexts in South Africa. This is in order to extend current understandings of social, cultural, and gender factors that shape men's SRH perceptions and practices, including those related to HIV.

In accounting for various constructions of masculinities, our study sought to avoid the unitary and generally negative characterization that often marks HIV/AIDS-related studies of men (Morrell *et al.* 2012). Connell's (1995) framework of hegemonic masculinity provides an avenue to deconstruct such homogenous understandings. Hegemonic masculinity identifies socially sanctioned norms that legitimize and privilege certain expressions of masculinity, such as being unemotional and aggressive. These ideal constructions of masculinity not only establish and maintain men's power over (some) women, but can also produce hierarchies between men, since men who do not adhere to dominant norms of masculinity are often marginalized by both sexes (Jewkes & Morrell 2010). The concept recognizes that not all men have equivalent privileges and power since factors including socioeconomic class, race, and sexuality can oppress men and impact on the feasibility of attaining a masculine ideal (Morrell 2007) and also appreciates that the most valued forms of masculinity differ depending on the social, historical, and cultural environment. This contextual nature of masculine norms means that they can and do change (Hearn, 2004).

There have been several criticisms of the concept of hegemonic masculinity, particularly the tendency to apply it in ways that portray men as either conceding to dominant gender norms or being marginalized by them. This common portrayal of men is limited as various studies suggest that men can hold multiple social identities simultaneously in a particular social context, or within a certain group (Frosh, Phoenix & Pattman 2002). Some scholars on masculinity have problematized the concept of hegemonic masculinity for giving insufficient attention to the production and maintenance of dominant masculine norms *by women* (Hearn 2004). It has been argued that masculinity studies tend to construct women as homogenous with little attention to differences, including race, class, and sexual orientation (Macleod 2007); moreover, research in the field rarely includes inputs from women. Through the use of men and women's sexual history narratives across various racial and cultural groups, the research seeks to contribute to the literature on hegemonic masculinity by appreciating the diversity in perceptions and expressions of masculinity, and how hegemonic male norms are constructed by both men and women.

We asked men and women to talk about their unique sexual life stories—their expectations, desires, perceptions of HIV risk, reproduction and sexual practices, and the meanings they attached to these. Narrative studies allow individuals to discuss their subjective experiences and understandings of their social norms and beliefs. According to Atkinson (1998), a life history refers to narration of one's life experience whereby one highlights the most important aspects in relation to the domain of inquiry. Connell and Messerschmidt (2005, 852) proposed that, ‘the careful analysis of life histories may reflect different hegemonic masculinity and also hold seeds of change’. Although the life history method is central to Connell's (1995) views on the complex manner in which masculinity is constructed, only a small portion of the research on masculinities over the past two decades has rested on a narrative approach (Wedgewood 2009). This study engages with the narrative methodology to access social constructions of masculinity, how these may influence men's SRH practices and care-seeking, and what implications the narratives hold for promoting HIV risk reduction among men.

The concept of hegemonic masculinity has generated a range of understandings of men's susceptibility and response to HIV risk, as well how to promote HIV prevention through transforming gendered socio-behavioral norms. Dominant constructions of masculinity in South Africa, including norms that promote men's engagement in multiple concurrent partnerships and sexual entitlement to women, have been shown to contribute to men's risky sexual practices and poor health-seeking behaviors (Hunter 2005; Jewkes & Morrell 2010; Lindegger & Quayle 2009). Society's often routine depiction of men as invulnerable, and a general perception that SRH clinics are women's domain, also play a role in hindering men from acknowledging health risks and seeking SRH care, including HIV testing (Dwadwa-Henda, Mfecane, Phalane, Kelly, Myers & Hajiyiannis 2010; Peacock, Stemple, Sawires, Sharif & Coates 2009). Men are more likely than women to delay anti-retroviral treatment (ART) and die of AIDS as a result (Mills, Ford & Mugyeni 2009; Shand, Thompson de-Boor, van den Berg, Peacock & Pascoe 2014). Research has also shown how men's risky sexual practices are strongly influenced by the social conditions in which they live and the associated dominant masculine repertoires. For example, in some study settings, HIV positive heterosexual men have been found to have lower levels of education and greater social vulnerability (Sandfort, Orr, Hirsch & Santelli 2008). In certain contexts, men's poor health-seeking behavior is exacerbated by poverty, for instance when men have to migrate for work, or work for long hours with limited time to seek healthcare even if it is available (Campbell, 1997). Wood and Jewkes (1997) documented how men in a resource-limited setting characterized by fatalism and violence, used misogyny and higher risk sexual practices to gain status and control over women. These studies show the interactions between individuals, community, and society that contribute to the gendered construction of men's SRH perceptions and practices. In addition, their insights helped to shape our study conceptualization, design, data collection, and analysis.

## Methods

The research used a qualitative narrative approach through two data collection methods: individual in-depth interviews (IDIs) to explore sexual life history narratives and focus-group discussions (FGDs) to further investigate SRH themes that emerged in the narratives in a group context.

## Participants

Sexual history narrative IDIs were conducted with 50 men and 25 women, and 10 FGDs were conducted with men, in 6 sites across 5 provinces in South Africa: a small town Grahamstown and a rural village Coffee Bay in the Eastern Cape; Cape Town, a city in the Western Cape; Pietermaritzburg, a city in KwaZulu-Natal; Nelspruit, a town in Mpumalanga; and Johannesburg, a major city in Gauteng. Although it was vital to have female participants share their stories about men's SRH and probe their perceptions and experiences of the gendered aspects of men's sexuality, the primary focus of the study was to assess the relationship between hegemonic masculinity and *men's* SRH. Therefore, we included a larger sample of male participants in the study. At each site (apart from Coffee Bay), the research was conducted in both the urban center and nearby rural areas. There is a wealth of research that links patterns of HIV prevalence to poverty and inequality (see Farmer 2005); therefore, to account for socioeconomic inequities, the research was carried out in the poorest (Eastern Cape and Mpumalanga) and wealthiest (Gauteng and Western Cape) provinces in the country. KwaZulu-Natal and Mpumalanga provinces also have the highest HIV prevalence rates in the country, with an HIV prevalence of 27.6% and 26.0%, respectively, among individuals in the 15–49-years age group in 2012 (Shisana 2013), emphasizing the importance of including these two provinces in the study. Conducting the study in diverse cultural, economic, and social contexts allowed the inclusion of participants across an array of different demographic categories. This is particularly important in the South African context, which is an extremely diverse country with 11 official languages (Anderson, Beutel & Maughan-Brown 2007). Table 1 presents the age, sex, language, race, and residential demographic breakdown of participants. To appreciate differences in SRH perceptions and behaviors across the lifespan, IDIs were conducted with men and women in 3 age categories at each site: 55 years and above, 25–54, and 18–24 years. To probe in greater detail the social issues that arose in narrative IDIs, 10 FGDs (2 in each province) were conducted with male participants from similar cultural backgrounds and allocated into 2 age groups (18–24 and 25–55 years). Table 2 presents the demographic background of FGD participants. Efforts were made to recruit heterosexual male and female participants to assess how norms of masculinity are constructed and maintained by both men and women in and through heterosexual relationships.

**Table 1.** Demographic background of interview participants.

Participant #	Sex	Age group (years)	First language	Race	Location
1	Male	18–24	isiXhosa	Black	Grahamstown (rural)
2	Male	18–24	isiXhosa	Black	Grahamstown
3	Male	25–55	isiXhosa	Black	Grahamstown
4	Male	25–55	isiXhosa	Black	Grahamstown
5	Male	25–55	Afrikaans	Colored	Grahamstown
6	Male	25–55	Afrikaans	Colored	Grahamstown
7	Male	25–55	English	Asian	Grahamstown
8	Male	25–55	Afrikaans	Colored	Grahamstown
9	Male	55+	English	White	Grahamstown
10	Male	55+	isiXhosa	Black	Grahamstown (rural)
11	Female	18–24	Afrikaans	Colored	Grahamstown
12	Female	25–55	English	White	Grahamstown
13	Female	25–55	Afrikaans	White	Grahamstown
14	Female	25–55	isiXhosa	Black	Grahamstown
15	Female	25–55	isiXhosa	Black	Grahamstown (rural)
16	Male	25–55	isiXhosa	Black	Cape Town
17	Male	18–24	isiXhosa	Black	Cape Town
18	Male	18–24	Afrikaans	Colored	Cape Town (rural)
19	Male	25–55	Afrikaans	Colored	Cape Town
20	Male	55+	Afrikaans	White	Cape Town
21	Male	25–55	English	White	Cape Town
22	Male	18–24	Afrikaans	Colored	Cape Town
23	Male	55+	Afrikaans	Colored	Cape Town
24	Male	18–24	isiXhosa	Black	Cape Town
25	Male	25–55	isiXhosa	Black	Cape Town
26	Female	25–55	Afrikaans	Colored	Cape Town
27	Female	55+	Afrikaans	Colored	Cape Town (rural)
28	Female	25–55	English	White	Cape Town
29	Female	25–55	isiXhosa	Black	Cape Town
30	Female	55+	isiXhosa	Black	Cape Town (rural)
31	Male	18–24	isiZulu	Black	Pietermaritzburg (rural)
32	Male	25–55	isiZulu	Black	Pietermaritzburg
33	Male	25–55	isiZulu	Black	Pietermaritzburg (rural)
34	Male	55+	isiZulu	Black	Pietermaritzburg (rural)
35	Male	18–24	Sesotho	Black	Pietermaritzburg
36	Male	25–55	Sesotho	Black	Pietermaritzburg
37	Male	25–55	English	Indian	Pietermaritzburg
38	Male	55+	English	Indian	Pietermaritzburg
39	Male	18–24	English	White	Pietermaritzburg
40	Male	25–55	Afrikaans	Colored	Pietermaritzburg
41	Female	25–55	isiZulu	Black	Pietermaritzburg (rural)
42	Female	25–55	isiZulu	Black	Pietermaritzburg
43	Female	25–55	Afrikaans	Colored	Pietermaritzburg
44	Female	18–24	English	Indian	Pietermaritzburg
45	Female	25–55	seSotho	Black	Pietermaritzburg
46	Male	18–24	isiZulu	Black	Johannesburg
47	Male	25–55	isiZulu	Black	Johannesburg
48	Male	18–24	sePedi	Black	Johannesburg
49	Male	25–55	sePedi	Black	Johannesburg
50	Male	25–55	seTswana	Black	Johannesburg
51	Male	55+	seTswana	Black	Johannesburg (rural)
52	Male	25–55	seSotho	Black	Johannesburg
53	Male	25–55	English	Indian	Johannesburg
54	Male	18–24	Afrikaans	White	Johannesburg
55	Male	18–24	English	White	Johannesburg
56	Female	55+	English	White	Johannesburg
57	Female	25–55	isiZulu	Black	Johannesburg
58	Female	18–24	seTswana	Black	Johannesburg (rural)
59	Male	18–24	isiXhosa	Black	Coffee Bay (rural)
60	Male	25–55	isiXhosa	Black	Coffee Bay (rural)
61	Male	55+	isiXhosa	Black	Coffee Bay (rural)
62	Female	25–55	isiXhosa	Black	Coffee Bay (rural)
63	Female	55+	isiXhosa	Black	Coffee Bay (rural)
64	Male	18–24	siSwati	Black	Nelspruit (rural)
65	Male	25–55	siSwati	Black	Nelspruit
66	Male	25–55	Afrikaans	Colored	Nelspruit
67	Male	18–24	xiTsonga	Black	Nelspruit (rural)
68	Male	25–55	xiTsonga	Black	Nelspruit (rural)
69	Male	18–24	isiZulu	Black	Nelspruit
70	Male	25–55	seSotho	Black	Nelspruit
71	Female	25–55	sePedi	Black	Nelspruit
72	Female	25–55	siSwati	Black	Nelspruit
73	Female	25–55	xiTsonga	Black	Nelspruit (rural)
74	Female	18–24	xiTsonga	Black	Nelspruit (rural)
75	Female	25–55	seSotho	Black	Nelspruit

**Table 2.** Demographic background of focus-group participants.

Focus group #	Gender	Age group (years)	First language	Race	Site
1	Male	18–24	isiXhosa	Black	Grahamstown
2	Male	25–55	isiXhosa	Black	Coffee Bay (rural)
3	Male	18–24	xiTsonga	Black	Nelspruit (rural)
4	Male	25–55	siSwati	Black	Nelspruit
5	Male	25–55	isiXhosa	Black	Cape Town
6	Male	18–24	Afrikaans	Colored	Cape Town
7	Male	25–55	isiZulu	Black	Pietermaritzburg
8	Male	25–55	English	Indian	Pietermaritzburg
9	Male	25–55	seTswana	Black	Johannesburg
10	Male	18–24	sePedi	Black	Johannesburg

A sample of 75 individuals is large by qualitative standards and sufficient to explore a wide range of perceptions, experiences, and practices, within a wide range of contexts. For resting on a qualitative approach, this research does not attempt to make generalizable conclusions about a particular age, social or cultural group, or context. Rather, we aimed to collect adequate data from a variety of people in different contexts to enable an identification of key emergent themes, insights, and understandings that could contribute to extant understandings of the social construction of masculinities that influence men's SRH.

## Recruitment

Interviewees were recruited through community contacts who could establish effective rapport with the participants—a critical consideration given the sensitive and personal nature of the topic. Community contacts were sourced through two non-governmental organizations: Centre of AIDS Development, Research and Evaluation (CADRE) (in Western Cape, Eastern Cape, Gauteng, and KwaZulu-Natal), and Sonke Gender Justice (in Mpumalanga and Western Cape). Each community contact was given R100 (∼US$10.00) for every participant recruited as reimbursement for the associated costs of transport and communication. Community contacts were required to have experience conducting recruiting participants for research and to be well acquainted with a rural and urban community in their respective study sites.

The first author distributed a project information sheet explaining the project purpose, the benefits, and risks of participating to community contacts at each study site. The contact person then prepared a list of people who matched the selection criteria of age categories, gender, race, and environmental backgrounds and discussed the list with the first author to ensure that the criteria were met. After explaining the study to eligible potential participants, the community contact notified those who were willing to participate that they would be contacted by the first author to set up a suitable time and venue for conducting the IDIs and FGDs. Working from initial contacts, snowball sampling was used to recruit further participants. An advantage of this sampling technique is that it enables the inclusion of otherwise hard to reach participants.

## Procedure

Data collection occurred between July 2010 and December 2011. Participants signed informed consent including having their interviews audio-recorded, and were given R100 (US$ 10.00) as a token of appreciation for their time and to reimburse them for transport costs incurred. Same-sex interviewers conducted IDIs and FGDs in the language preferred by each participant. The first author conducted interviews in English with female participants. Experienced qualitative researchers conducted all other IDIs and FGDs. IDI guides were designed to enable interviewers to probe similar issues with participants. The guides comprised key questions and probes designed to encourage the telling of sexual history narratives: they began by soliciting accounts of early knowledge of sex and sexual experimentation, and then asked about the range of sexual relationships and reproductive health choices through adulthood. Probes were used to explore perceptions of masculinity, risks of HIV, and other sexually transmitted infections (STI). FGD guides were designed to further explore SRH-related themes that emerged from the narratives in a social context. All interviews were audio-recorded, transcribed, and where necessary, translated into English. After transcription, recordings were deleted to ensure confidentiality of data, transcripts were kept on secure password-controlled computer that only the research team had access to, and all real names were erased and changed to pseudonyms.

## Data management and analysis

The data were analyzed using a thematic approach. Thematic analysis aims to understand an issue by revealing the prominent themes at various levels in a text in order to provide a holistic and nuanced account of data (Attride-Stirling 2001). First, analysts read the raw data several times to familiarize themselves with content and meanings. Data were then analyzed for content using nVivo 7 qualitative data management software. Text segments were assigned basic codes, and these codes were organized into major trends and crosscutting themes. Coding was regularly discussed between the first author, researchers from CADRE, and the contracted fieldworkers (who conducted the interviews) to achieve conceptual alignment on existing and emerging codes. An overall interpretation of the findings of the study was formulated, showing how thematic areas relate to one another and in relation to the research questions. Illustrative quotes relevant to each theme were extracted from the raw data. These reflect dominant and divergent perceptions of masculinity that emerged from the participants' narratives. All illustrative quotes include demographic details of the participants; names used in this text are pseudonyms.

## Enhancing research quality and rigor

Several techniques were used to enhance the rigor of the research. Research foci and questions were informed by a comprehensive review of the literature. As the study aimed to generate insights and add to extant knowledge in the field, the sample size was adequate to achieve these ends. In addition, the use of snowball sampling diminished the risk of selection bias that may have arisen through relying only on community contacts to recruit participants. All fieldworkers were appropriately trained, sensitized, and supported. They comprised multilingual men and women so that they could conduct interviews in participants' preferred languages and between persons of the same sex. These factors are important given the personal and intimate nature of the research and the interest to asses how participants perform their IDIs with members of the same sex. Audio-recording of interviews ensured that data were correctly and precisely captured, without subjective filtering of information. More than one analyst oversaw data analysis and the coding process, which allowed for the interrogation of interpretations as well as for emerging interpretations and insights. Finally, ethical approval granted by the University of Cape Town's Research and Ethics Committee in the Faculty of Health Sciences (REC REF 115/2011) ensured that the research design complied with human rights and dignity. Given the sensitive and potentially evasive nature of detailing private, and highly stigmatized, sexual stories to interviewers, opportunities for debriefing were provided. Confidentiality and anonymity were ensured for IDI participants; while FGD participants were made aware that these could not be ensured, discussions began with participants pledging not to divulge anything said in the group to anyone outside of the group.

## Findings

In this section, findings from the narrative IDIs and FGDs are presented. Analyses revealed three overarching themes that capture the social construction of masculinity as it interacts with men's SRH perceptions and practices. These are: (1) preventive behaviors against HIV, (2) HIV testing, and (3) STI/STI awareness and prevention.

## Preventive behaviors against HIV

For the majority of participants—who were generally well informed about HIV risk and prevention—there were several factors related to hegemonic notions of masculinity that impeded them from assimilating SRH messages and translating their knowledge into safer sex practices. Many participants noted a disjuncture between what they felt they should be doing and their lived sexual practices. Some men expressed a lack of sexual ‘self-control,’ a trait that is often associated with dominant norms of male sexuality. One man admitted:

I don't have self-discipline. I'm trying but not hard enough. I told you about my friend that died of HIV. Even so, I can still have sex with a girl without a condom and I can sleep with several girls without it. It showed me that I was living in denial. (Melisizwe CPT, M isiXhosa-speaking 25–55)

An additional barrier to safer sexual practices was perceptions among young men that they were not at risk of HIV infection or were ‘immune’ or felt invincible to the virus:

To be honest, I thought I'm young, I'm just going to have sex and it's just like playing. So we didn't even think to take precautions. (Khulani, JNB, M Zulu-speaking 18–24)

## Barriers and facilitators to male condom use

While male condoms were regularly perceived of as the most preferred, effective, and accessible prevention method against HIV, participants acknowledged that there were still many barriers to their use. Commenting on the diversity of men's safer sexual practices, a female participant stated:

I think most men do carry [condoms] and then some don't. It's either because they don't think they have to or that it's the women's responsibility or they just don't really think about it, they just don't care. But I think most of the men out there do actually carry protection with them just in case. (Lesley, CPT, F English-speaking 25–55)

Both men and women discussed how a reluctance to use condoms was more common among men. Several women related a sense of responsibility in encouraging men to use condoms. As one woman said: ‘Guys are stubborn, so it's our duty to encourage them to use a condom’ (Bina, Rural PTZB, F isiZulu-speaking 25–55). Participants mentioned numerous barriers to men's consistent and regular use of condoms, including a feeling that sex needed to be consummated in the ‘heat of the moment’. One woman reported on the common difficulty for men especially to interrupt sex in order to put on a condom ‘they are kissing and stuff and they are caught up in the moment of making love. And if there is no condom he just goes. I will face the consequences’ (Gabby, NELS, F Xitsonga-speaking 18–24). Notions of men having an incontrollable sexual urge were said to hinder men's condom use. One woman recalled a man who assumed a sense of fatalism rather than opting to use condoms with his multiple partners:

He loves girls. He has three women already. He tells you straight, ‘Why must I use a condom?’ Just like that. He just tells you straight off, ‘If I die, I die anyway. (Abigail, PTZB F Colored Afrikaans-speaking 25–55)

Another key barrier for men to use condoms was anxiety about decreased sexual performance:

When I use a condom I can go only five minutes a round, but when I don't have a condom I can go fifteen minutes a round. (Daegan, NELS, M Xitsonga-speaking 25–55 FG)

In a similar vein, another participant commented on the sexual desire for ‘flesh-to-flesh sex’ stating ‘you can't have a lollipop with the wrapper on’ (Simo CPT, M isiXhosa-speaking 25–55). Some men expressed their concern that women too may experience less sexual pleasure when they use condoms. As one man stated: ‘A woman will never feel pleasure with plastic. They won't need us’ (Tembe, Rural NELS, M SiSwati-speaking 25–55 FG). Many of the men mentioned their dissatisfaction with the quality of Choice^©^ brand condoms, which are freely issued to the public by the South African government. They said they felt these condoms were too small and inadequately lubricated, and as a result could cause them lose their erections.

The belief among some men that condoms are not effective against HIV transmission could impact negatively on their use. One man stated: ‘condoms are a Western tool. They do not work here in Africa’ (Duane, Rural NELS, M Colored Afrikaans-speaking 25–55). Some men also considered it healthy for women to have sperm enter them as they believed that there were vitamins in sperm and this discouraged condom use. Several participants, especially younger men, reported that condom use was less likely when they consumed too much alcohol. Women participants in particular spoke of difficulties negotiating condom use when their partners were drunk. The issue of trust in more established relationships hindering condom use appeared often in the narratives:

When you are with someone you use a condom the first time you have sex with them, second time, by the third time you are used to that person and you almost trust them. No one uses a condom longer than that. And that's when you can still die. (Tembe, Rural NELS, M SiSwati-speaking 25–55 FG)

However, there was some divergence from this with some men reporting use of condoms in all types of relationships. To protect themselves and their partners from acquiring STIs, including HIV. One man reported that his attitude toward condom use changed because of his developed awareness of HIV risk:

At a very tender age I was in a stable relationship so I never had opportunities to use these condoms much, and I realized I enjoy having sex without a condom better than I do with a condom. But only now I prefer to use condoms cause I am now well aware that I might just lose my life. (Eugene, CPT, M Colored Afrikaans-speaking 25–55)

Some participants noted a growing trend in condom use, particularly among young men, due to improved awareness of the seriousness of HIV, especially in the context of knowing someone who died of AIDS. As one woman commented:

Nowadays it is rare that you find a guy saying I will have sex without a condom. Now more guys understand. Especially the young ones because they now see the reality, the side effects. Sometimes you find a family member dies of HIV and you realize that this thing is real. (Bina, NELS, F seSotho-speaking 25–55)

The notion of taking personal responsibility for condom use arose several times in the data, along with the idea of condom use as a habit that needs to be fostered for the good of self and others. One male participant said:

I do not want to teach myself to get used to not using a condom so that I can put other people's lives in danger. (Malik, GTOWN, M isiXhosa-speaking 25–55)

Another participant asserted his responsibility for condom use to avoid the possibility of transmitting HIV to his partners:

I was not really worried about me. I was worried about other people, of putting other people's lives in danger. The most depressing thing is to think that other people can die because of you. Even today that is the reason that makes me use a condom—as a responsible person. (Simo, GTOWN, M isiXhosa-speaking 25–55)

Participants reported the stigma for women to buy, carry, and initiate condom use, with men generally being expected to carry condoms and initiate their use. Yet, there was also much divergence from this norm. As one man said:

It would be good if they [women] kept condoms in their purses. If she meets a guy she mustn't be dependent on a guy having it or being a proactive one. Because we as guys can excuse things. We can sometimes do it purposely and say 'I don't have a condom' when you know you have got. So if she's going to sleep around, there must be a condom in her pocket. (Bongani, CPT, M isiXhosa-speaking 25–55)

This may reflect the fact that attitudes are changing in relation to women buying and carrying condoms, which has for many years been seen as an indication of promiscuity. Indeed, several male participants reported being supportive of women who shared responsibility for condom use, for instance: ‘With my steady girlfriend I will use a condom. That's the agreement between the two of us. She is a strong woman; she can stand on her own feet.’ (Ndumiso, JNB, M isiZulu-speaking 18–24)

## HIV counseling and testing

HIV counseling and testing (HCT) seemed to be highly accessible, apart from some rural areas, and several participants reported having an HIV test regularly, although this was much more common among women than men. Men were less likely than women to access SRH clinics and thus learn about HIV testing through this avenue. Several men mentioned that they felt excluded from clinics offering HCT for being perceived to be a women's domain or an area to which they were not welcomed. For many men, a barrier to testing for HIV was said to be linked to a reluctance to seek help until very ill. The possibility of testing HIV positive frequently induced feelings of fear and could deter people from testing, as highlighted by one male participant:

Cause man I mean I'd rather die not knowing I'm gonna die than die knowing I'm gonna die on a certain day. I think that's gonna get me even more skinny. I'm gonna get sick. If I don't know, I think I'll be better. (Sipho, JNB, M isiZulu-speaking 18–24)

Several participants reported that women were often instrumental in encouraging their male partners to test. One woman's story illustrates this:

Women encourage men since I am the one who goes to the female clinic and I get tested and my partner didn't go to the female planning. So when they teach me about HIV and AIDS, it is then my turn to tell him. Women are helping men. (Bongekile, Rural NELS, F xiTsonga-speaking 18–24)

Many women recalled becoming aware of the importance of HIV testing from contraceptive or antenatal clinics they had attended, which were generally less accessed by men. Several men said that they overcame their fear to test when their partners became pregnant. The following comment reveals the interplay of men's perceptions and attitudes toward different types of relationships with women in relation to trust and worth that could influence SRH practices, including HIV testing:

There is a difference between makwapeni (side partners) and my wife. Because my wife, I have had some HIV tests with her. And then I trust her too much. And I know she is faithful even though myself I am not faithful. But with makwapeni I don't give them love. I always use condoms with them. (Rhulani, NELS, M xiTsonga-speaking 25–55 FG)

However often in more established relationships, sex was perceived of as less risky and couples reported having unprotected sex without testing for HIV. Nonetheless, those who reported having had an HIV test tended to report adopting safer sexual behavior as a result. As one man said:

Yes you know before I got tested I wasn't using condoms. After I tested my status, I started using condoms. When I realised I was negative I wanted to stay that way. (Ntokoto, JNB, M xiTsonga-speaking 25–55 FG)

There were also narratives of individuals who tested for HIV regularly to check that they maintained their negative HIV status. One man spoke of the respect he gained within his community for regular testing:

Even now the people respect me when I go to these things. And they say this man checks himself! Because I decided to go and test when I heard about it. And I test regularly. (Yongama, RURAL CBAY, M isiXhosa-speaking 55+)

Another man reported continuing to have unprotected sex with his girlfriend after she had been diagnosed as HIV positive. He feared that he may have been responsible for infecting his partner and assumed that because she was HIV positive he was also HIV-infected. When he later tested HIV negative, his relief at being uninfected and not being responsible for his partner's infection, motivated him thereafter to take responsibility for safer sex by using condoms consistently: ‘I cannot, if I love someone, put them at risk by not using a condom.’ (Malik, GTOWN, M isiXhosa-speaking 25–55)

## STI/STD awareness and prevention

There seemed to be a general consensus among men and women that since STIs were curable they were not as worrying as HIV, and thus did not induce as much fear. One man stated:

You don't care if you have an STI. You just want to f*** the girl, period. There is no problem what she gets. That's the mindset many men have: It's not my problem. It doesn't matter how many warts I have around the penis, as long as I don't get AIDS. (Eugene, CPT, M Colored Afrikaans-speaking 25–55)

Overall women seemed to have more knowledge of STIs than men, including their signs and symptoms. They had mostly learned about these from attending reproductive health clinics. One male participant reported being unsure how to determine if his partner had an STI and thought that having ‘small pimples' on her face was a sign of an STI. Another man's statement highlights misconceptions held about the signs or effects of STIs, saying: ‘use a condom because otherwise something will happen and your private parts will go green.’ (Mustapha, JNB, M Indian English-speaking 25–55)

One man (Kefentse, NELS, M xiTsonga-speaking 25–55) recalled how he once had an STI but waited five days to go to the clinic because as a man he felt pressured to not seek healthcare but instead to endure pain. He only went to a clinic when the pain became unbearable, where he was given medicine that healed him. His story is echoed in those of several other male participants who were compelled to go to the clinic for STI treatment, and in doing so had an HIV test, learned about HIV prevention, and thereafter adopted safer sex practices. One man reported that men tended to not consider themselves as being at risk until they contracted an STI and that this lack of experience hindered safer sexual practices:

It's a pity because one still engages in unprotected sex. I guess it's a weakness of some of us men. … maybe the outcome for me to actually still engage in [unprotected] sex would have not been the same if I had actually experienced it myself. Seeing something there and actually experiencing it yourself are two different things. (Bafana, Rural PTZB, M isiZulu-speaking 25–55)

## Discussion

### Barriers and facilitators to men's SRH

The narrative excerpts show variation in SRH practices, reminding us that patterns of interactions between perceptions, knowledge, practices, contexts, and personalities are complex and, as Hearn (2004) points out, contingent on different circumstances. A notion that weaves throughout the narratives is that men tend to lack ‘sexual self-control,’ and/or are sexually invincible, which could undermine their HIV risk perceptions, impeding their engagement in HIV prevention behaviors. This is in keeping with findings documented in other studies (Anderson *et al.* 2007; Napper & Fisher 2012). Men's reluctance to use condoms being linked to feelings of insecurity in their ability to sexually please their female partners is also in reflected in other findings (Schneider, Cockcroft & Hook 2008). This sense of vulnerability and concern goes against normative injunctions of men as sexually confident and controlling, and uninterested in women's sexual pleasure. Several men, particularly young men, reported that alcohol consumption acted as a barrier to practicing safer sex, supporting evidence from other studies that heavy drinking may lead to sexual inhibition (Parker & Borwanker 2012; Pettifor, Measham, Rees & Padian 2004), and that alcohol use is higher among men than women in all age groups, provinces, and populations in South Africa (Peacock *et al.* 2009). Consistent with other studies (Corbett, Dickson-Gormez, Hilario & Weeks 2009; Cusick & Rhodes, 2000) and findings from this study elaborated on elsewhere (Stern & Buikema, 2013), some men were less likely to use condoms in more established relationships, which were perceived to be less risky than casual relationships in terms of STI transmission. However, other male participants reported taking responsibility for their own and their partner's sexual health and consistently using condoms in all types of relationships. Despite the reported stigma of women carrying and initiating condoms, a number of men supported the right of women to share in SRH decisions with their male partners and encouraged women to exercise control over their sexual health by carrying condoms and initiating their use. These attitudes may reveal an emergent trend toward a more gender-equitable masculinity, one that respects the sexual agency of women.

In this study, more women than men recalled testing for HIV and testing regularly. This has also been reported in other studies (Cornell, McIntyre & Myers 2011; Lynch, Brouard & Visser 2010). Nevertheless, hegemonic masculine norms encouraging men to see themselves as invincible acted as barriers to HIV testing among men. This disparity also indicates a need for men to be educated about the importance of knowing their HIV status, and SRH services to better cater to men's HIV testing needs (Shand *et al.* 2014). This can include enhancing HIV service accessibility in communities, in light of evidence that men are more inclined to use community-based HIV services (Shand *et al.* 2014). HIV interventions seeking potential entry points to promote men's HIV testing and early attendance at SRH care could build on women's role in encouraging men's health-seeking behavior, which was positively documented in the study. The fact that women could encourage their male partners to test for HIV also presents opportunities, where feasible, for couples' HIV testing to reduce HIV risk behavior. This approach is in keeping with recommendations from other studies (Kelly, 2009) and aligns with South Africa's new testing policy and that of the WHO (2012), which seek to promote more widespread HCT for couples. Some men's increased willingness to test for HIV during a partner's pregnancy could be utilized to encourage greater male partner participation in prevention of mother-to-child HIV transmission programs. As Morrell and Jewkes (2011) note, men's engagement in carework and fatherhood can fuel a change in gender identity and value transformation, and promoting this can advance gender equity. There was some evidence that individuals testing HIV negative encouraged them to maintain this status through safer adopting sexual behavior and re-testing their HIV status regularly. Many of the narratives indicate that individuals who know they are living with HIV take steps to prevent passing the virus onto their sexual partners. This counters an oft-repeated urban legend that HIV positive individuals who are unable to deal with the implications of their status seek to deliberately pass on the virus to others. This has been sensationalized by media reports of a few such cases in some developed countries.^1^

Fear as a deterrent to higher risk sexual practices was a common theme in the narratives; with men and women tending to be more concerned about HIV infection than other STIs, for being perceived as curable. Women had a greater awareness of STIs than the men did, and predominantly learned about STIs at SRH clinics. Fewer women discussed actually receiving treatment for an STI than men. This could reflect the fact that STIs are more commonly asymptomatic in women than in men and therefore more frequently go untreated (Cooper, Morroni, Orner, Moodley, Harries, Cullingworth, *et al.* 2004). In line with other findings—and in keeping with a common cultural construction of masculinity in South Africa—many of the male participants perceived contraception and SRH care as ‘women's business' (Kelly, 2009; Peacock *et al.* 2009). Men commonly described being reluctant to visit a clinic for STI treatment until their infection became severe, illustrating how hegemonic norms that expect men to not show weakness through illness can negatively impact on men's health-seeking behaviors. Colvin, Robins and Leveans (2010) also found that beliefs that real men can ‘tough it out’ translated into men's failure to access care and other forms of support on time. On a positive note, many of the men who were tested and treated for STIs reported that as a result they became more motivated to practice safer sex. This demonstrates the continued need to promote information about the links between HIV and other STIs, and also to ensure that SRH services are male-friendly and accessible.

Insights on differences between rural and urban participants' SRH awareness confirm the value of conducting research in sites that are diverse geographically, economically, and culturally. Rural participants noted that SRH services, including the availability of HIV testing and condoms, were less accessible in their areas. Together with difficulties in accessing SRH and HIV services, misconceptions about HIV, STIs, and condoms were more pronounced in the rural areas.

Finally, the findings indicate how some women are complicit with hegemonic masculine norms and notions that impact negatively on SRH. For instance, some women endorsed the idea that men are unable to control their sexual impulses and cannot be expected take responsibility for contraception use. The data also attest to the fact that women can have a positive influence on men's SRH behaviors, such as setting and adhering to shared boundaries in relation to condom use, and encouraging men to test for HIV. This highlights the importance of understanding women's perceptions of men's sexuality and SRH, as these notions can play a role in challenging and changing hegemonic norms of masculinity, or reinforcing and reproducing them (Hearn, 2004). Moreover, as Mfecane (2013) argues, women's agency around sexual decision-making must be understood within gendered power inequalities in South Africa. Women's perspectives from this study are elaborated on further in Stern and Buikema (2013) and are not the primary focus of this paper.

## Limitations

Narratives of sexual history are of necessity retrospective and subject to memory, which can be prone to being selective. It is quite possible that there were discrepancies between perspectives about gender norms and values as revealed through the narratives and the lived experiences of men and women due to social desirability bias, which is especially pertinent around the highly stigmatized topic of sexuality. Nonetheless, many participants showed great interest in the interview topics and spontaneously commented on the value of discussing their sexual histories.^2^ If participants had been interviewed on more than one occasion, their narratives may have changed. For example, Martin (2010) found when he conducted a series of longitudinal sexual narrative interviews with a cohort of young Vietnamese men, participants often modified or rejected aspects of their previous narratives in subsequent interviews. Data translated into English for this study did not undergo a formal process of back-translation, as doing so with many interviews and FGDs was prohibitive. This may have affected the accuracy of the raw data.

The authors were aware of their identities as white, middle-class, urban, women exploring the experiences of South African men and women from a variety of racial, cultural, economic and geographic backgrounds. To minimize a biased interpretation of the findings, the authors opened the analysis process to inspection and verification by other researchers who conducted the IDIs and FGDs. All the authors were mindful about social and cultural norms in the analyses and presentation of the findings, and endeavored to be self-critically aware and reflexive of their assumptions and positions. This was particularly important given that some research in the area of HIV and masculinities has tended to demean African male sexualities, or be ignorant or contemptuous of African cultures (Ratele 2014). By including a diversity of men, this study also avoided a predominant focus on African male sexuality.

## Conclusion

Collecting sexual history narratives from men and women was an effective method to generate in-depth insights on men's SRH perceptions and practices and the underlying social constructions of masculinity that shape these. Our study adds value by privileging the diversity of men and women's SRH values, needs, and interests across a broad range of participants from diverse contexts. As Ntshebe, Pitso and Segobye (2012) indicate, this kind of information can be useful in shaping interventions that target men's SRH attitudes and behaviors, where they stand in the way of attaining healthy SRH and gender equity.

The narratives draw attention to the many ways that certain gendered constructions make men and women vulnerable to HIV, supporting the idea that HIV prevention programs should do more than attempt to alter individual sexual behaviors by addressing perceptions of masculinity and femininity that frequently underlie risky and gender inequitable sexual practices (Jewkes & Morrell, 2010). Indeed, a 2007 WHO review of 57 interventions that work with men in SRH found that gender-transformative programs that promoted shifts in gender norms and fostered more gender-equitable relationships between the sexes were more effective in encouraging safer SRH attitudes and behaviors than programs that were ‘gender sensitive’ or ‘gender neutral’ (Peacock & Barker 2012). The fact that several participants appreciated the process of telling their sexual history narratives points to unmet needs and limited opportunities to reflect on and discuss their sexual relationships and behaviors. The process of articulating one's sexual history can open a space for critical reflection on one's own SRH practices and attitudes and how these are shaped by gendered constructions. Having people narrate their sexual history stories is thus not only a research approach but also has potential as a form of SRH intervention.

A further value of the study is that the findings support the suggestions of leading authors on studies of masculinity (such as Hearn 2004 and Barker 2005) that factors and processes impacting on potentially unhealthy or risky masculine perceptions and behaviors are constructed within varied and multiple power structures and socio-cultural contexts. Ultimately, this study points to the value of sexual history narratives in more deeply understanding men's sexuality and SRH, and the social structures, meanings, and experiences that underlie it. Such insights are critical for better engaging men in HIV gender-transformative prevention efforts.

## Notes

1 Shilt's (1987) book ‘And the Band Played On’: Politics, People and the AIDS Epidemic (St. Martin's Press), which includes extensive discussion of a Canadian flight attendant Gatean Dugas's progression from HIV infection to death and his many sexual liaisons, received extensive media coverage around Dugas's ‘demonized’ role in spreading AIDS on the continent. Dugas was defamed in the press, particularly in Canadian media as disgracing the nation, despite the fact that he could not have known, as we do today, the facts regarding HIV transmission.

2 One man said:
  I would have to say I was hungry for an interview like this one. Just to get a chance to express myself, to get something out, because I didn't know this was part of what I am carrying all the years, so just to get something out made me feel much better. (Barry, CPT, M Coloured Afrikaans-speaking 18–25) A woman participant (Thabisa, JNB, F isiZulu-speaking, 25–55) added: I haven't spoken to my husband about this. There are many days that this heavy mood is still hanging onto me. And now I have opened up and I am so grateful that I found somebody to whom I could open up.

## Interview appendices

Codes for participant referencing

M = Male
F = Female
FG = Focus Group
CBAY = Coffee Bay
CPT = Cape Town, Western Cape
GTOWN = Grahamstown
JNB = Johannesburg, Gauteng
NELS = Nelspruit
PTZB = Pietermaritzburg, KwaZulu-Natal
R = RURAL
